# Cluster-randomised controlled trial of an occupational therapy group intervention for children designed to promote emotional wellbeing: study protocol

**DOI:** 10.1186/2050-7283-2-16

**Published:** 2014-06-23

**Authors:** Ema Tokolahi, Clare Hocking, Paula Kersten, Alain C Vandal

**Affiliations:** Auckland University of Technology, Centre for Person-Centred Research, AUT University, Private Bag 92006, Auckland, 1142 New Zealand; Centre for Person-Centred Research, Auckland University of Technology, Private Bag 92006, Auckland, 1142 New Zealand; Department of Biostatistics and Epidemiology, Auckland University of Technology, Private Bag 92006, Auckland, 1142 New Zealand; Health Intelligence & Informatics, Ko Awatea, Counties Manukau District Health Board, Private Bag 93311, Auckland, 1640 New Zealand

**Keywords:** Occupational therapy, Health promotion, Anxiety, Depression, Self-esteem, Participation, Wellbeing, Children, Schools

## Abstract

**Background:**

Symptoms of anxiety and depression are common in childhood, as are risk factors that undermine wellbeing: low self-esteem and limited participation in daily occupations. Current treatments focus primarily on modifying internal cognitions with insufficient effect on functional outcomes. Occupational therapists have a role in measuring and enabling children’s functional abilities to promote health and wellbeing. To-date there is no evidence for the use of occupational therapy as an intervention to promote mental health or increase self-esteem, participation and wellbeing in a preventative context. The aim of this cluster-randomised controlled study is to investigate the effectiveness of an 8-week occupational therapy group intervention (Kia Piki te Hauora) at reducing symptoms of anxiety and depression and improving self-esteem, participation and wellbeing in children aged 11–13 years.

**Methods/design:**

In this two-arm, pragmatic, cluster-randomised controlled trial, 154 children will be recruited from 14 schools. All mainstream schools in the region will be eligible and a convenience sample of 14 schools, stratified by decile ranking (i.e. low, medium, and high) will be recruited. Eight to twelve students aged 11–13 years from each school will be recruited by senior school personnel. Following consent, schools will be randomised to either the intervention or waitlist control arm of the trial. The study will employ a parallel and one-way waitlist-to-intervention crossover design. Each cluster’s involvement will last up to 19 or 31 weeks depending on allocation to the intervention or waitlist respectively. The primary outcome is symptoms of anxiety and secondary outcomes are symptoms of depression, self-esteem, participation in daily occupations and wellbeing. Outcome measurement will be repeated at baseline, post-intervention and again at 8–9 weeks follow-up. Planned statistical analyses will utilise repeated measures analysis of covariance. The primary analysis will be based on an intention-to-treat analysis set and include only parallel data. The crossover data will only be used in secondary analyses.

**Discussion:**

This is the first cluster-randomised controlled trial to investigate an occupational therapy intervention promoting emotional wellbeing in a non-clinical sample of children. Results will contribute to the limited evidence base for occupational therapists in this field and potentially support investment in these services.

**Trial registration:**

Australia/New Zealand Clinical Trials Register: ACTRN12614000453684.

## Background

Up to 10.5% of children aged 10–14 years reportedly experience anxiety that produces unhelpful internal cognitions and impacts on their participation in occupations, level of functioning and development (Costello et al., 2003; Zahn-Waxler et al., 2000). A significant relationship between anxiety and depression has been clearly identified (Costello et al., 2003; Silverman & Treffers, 2001) with anxiety disorders in childhood and adolescence shown to precede and predict later depressive disorders (Merrick, 1992; Zahn-Waxler et al., 2000). Consequently, the need for early intervention is vital (Friedberg et al., 2000).

Anxiety, participation and functioning are closely related. A child with symptoms of anxiety may present with numerous worries that may seem disproportionate or more pervasive than those expressed by their peers; they may be restless, easily fatigued, have difficulty concentrating or be reluctant to participate in the same activities as their peers (American Psychiatric Association, 1994). Participation can be defined as ‘involvement in a life situation’ or meaningful occupation, such as school or play (World Health Organization, 2007, p. 9). Merrick ( 1992) found increased mental wellbeing in children was related to increased levels of participation in occupations and Krupa et al. ( 2003) reported an imbalance in occupational participation created an increased risk for wellbeing and the onset of mental ill-health. Functioning, as defined by the World Health Organization (WHO) is a combination of the body’s physiological and anatomical structures and the ‘execution of a task or action by an individual’ that impact on an individual’s ability to participate in life situations (World Health Organization, 2007). Despite this well-documented relationship between anxiety, participation and functioning, evidence for interventions directly targeting functioning and participation, rather than cognitive processing errors alone, is limited (Vitiello et al., 2006).

We reviewed published experimental research that explored the range of preventative mental health interventions available for children (aged 7–19 years) in the school environment. The majority of interventions described demonstrated the effectiveness of Cognitive Behaviour Therapy (CBT) at modifying an individual’s internal cognitions (Bernstein et al., 2005; Dadds et al., 1997; Horowitz et al., 2007; Pössel et al., 2005). However, CBT has been found insufficient in effecting significant change when level of functioning was measured as an outcome (Vitiello et al., 2006).

Other common interventions reported were building social networks and skills for help-seeking (Aseltine & DeMartino, 2004; Eggert et al., 2002; Thompson et al., 2001) and developing life skills (Eggert et al., 2002; Thompson et al., 2001). A few studies reported interventions based on Interpersonal Therapy (Horowitz et al., 2007), physical activity (Bonhauser et al., 2005), and information processing (Pössel et al., 2005). All of the interventions reviewed were conducted with students in groups and ranged in duration from 1 to 2 hours per week over a period of 8 to 12 weeks. In all studies reviewed the intervention group was compared to a control group, with some studies also including comparisons to an alternative intervention or an attention-control group also (Bernstein et al., 2005; Eggert et al., 2002; Horowitz et al., 2007; Thompson et al., 2001). Most studies explicitly reported the use of cluster randomisation and a few indicated randomisation occurred at the individual level (Aseltine & DeMartino, 2004; Bonhauser et al., 2005; Horowitz et al., 2007). All studies reviewed involved repeated measures with an explicit or implied intention-to-treat approach to analysis. Blinding was not fully described for any of the studies; however some reported outcome assessors being blinded to condition allocation (Bernstein et al., 2005; Dadds et al., 1997; Horowitz et al., 2007) and many referred to preventing contamination between conditions. Nomination by a teacher was a common method for identifying participants.

General conclusions from these studies indicated experimental interventions were more effective at reducing the rates of symptoms of concern than control interventions; in the latter, symptoms typically increased (Dadds et al., 1997). Strong evidence was therefore demonstrated in favour of the need for preventative interventions. A common discussion point was late childhood as a key time for targeting preventative interventions and acknowledgement that universal interventions are typically not universally effective, with greater benefits being measured in those with greater needs at baseline (Dadds et al., 1997; Horowitz et al., 2007). Preventative interventions aimed at those with greater needs at baseline, where early or mild symptoms of a disorder are already present, have been categorised as ‘indicated’ (Neil & Christensen, 2009).

A viable intervention approach not yet investigated in the research literature is occupational therapy to promote mental health and reducing symptoms of mental ill-health in children. Occupational therapy’s unique contribution to health promotion includes: reduce risk factors and symptoms through engagement in occupation; providing skill development training in the context of everyday occupations; and providing training in adaptation to change and coping with adversity to promote mental health (not an exhaustive list) (AOTA Commission on Practice, 2001, 2008; Godfrey, 2000). Occupational therapists can assess how able a child is to participate in daily occupations outside of a standardised context and how frequently the child actually participates, and can use the child’s performance of developmentally appropriate occupations to promote health and wellbeing (Costello et al., 2003; Cramm et al., 2012; Zahn-Waxler et al., 2000).

The need for research validating the effectiveness of occupational therapy in primary health has been identified as essential (AOTA Commission on Practice, 2001; Nicholson, 2011). A literature search found one article that explored the role of occupational therapy in promoting wellbeing in a population exposed to a common trauma i.e. child survivors of war (Simo-Algado et al., 2002). To date, there is no evidence for the use of occupational therapy as an intervention to promote general mental health and increase self-esteem and participation in a primary health setting or preventative context.

### Aim and hypotheses

The aim of this study is to use the rigour of a cluster-randomised controlled trial to investigate the effectiveness of an indicated occupational therapy group intervention (Kia Piki te Hauora) at reducing symptoms of anxiety and depression and improving self-esteem and participation in children aged 11–13 years on completion of the intervention and the sustainability of any improvements after a follow-up period of 8–9 weeks.

#### Primary hypothesis

There will be a difference in post-intervention levels of child-rated anxiety between the intervention group and the waitlist control group.

#### Secondary hypotheses

- There will be a difference in post-intervention levels of self-, parent- and teacher-rated symptoms of anxiety and depression, child self-esteem and participation in typical occupations between the intervention group and the waitlist control group.
- There will be a difference in self-, parent- and teacher-rated symptoms of anxiety and depression, child self-esteem and participation in typical occupations between post-intervention and 8 weeks follow-up.
- There will be a relationship between change in participant knowledge about occupations, health and wellbeing between pre- and post-intervention and change in the other outcome measures.

## Methods

### Trial design

This is a two-arm, pragmatic, cluster-randomised controlled trial in which schools are the unit of randomisation. The trial will employ an open-label, repeated measures, parallel and one-way crossover design. Children will be naturally clustered by school and this design will prevent contamination between trial arms (intervention and waitlist). One-way crossover will occur when children allocated to the waitlist crossover to start receiving the intervention after a set time. The trial has been approved by the New Zealand Health and Disability Ethics Committees (14/NTA/13) and is registered with the Australia/New Zealand Clinical Trials Registry (ACTRN12614000453684) http://www.anzctr.org.au/default.aspx.

### Study procedures

#### Setting

All schools in the Auckland region, providing mainstream education to children in Years 7 and 8 will be invited to participate through a notice in a national teaching magazine. In New Zealand, Years 7 and 8 cater to the needs of children aged 11–13 years. A stratified sample of 50 of those schools will be sent personal invitations to participate. Schools will be stratified by decile ranking proportionate to their representation nationally (i.e. low, medium, and high): decile ranks are a measure of the socio-economic population from which a school draws its students and affects their funding (Ministry of Education, 2014). Fourteen schools will be recruited with stratification targets of 4 for each of the low and high deciles, and 6 for the medium decile. Senior staff from schools expressing an interest will be offered a meeting with the lead researcher who will explain the study process and obtain consent. This process will be repeated until all 14 schools have been recruited.

#### Participants

A sample of 10–12 students aged 11–13 years at each of the enrolled schools will be recruited by senior personnel from the school (e.g. Special Education Needs Coordinator, Principal). Selection will be based on the school personnel’s judgement of the child presenting with early symptoms of anxiety along with symptoms of depression, low self-esteem and/or poor participation in typical occupations. Participant information sheets and parental consent forms will be sent home with the children and collected back in by school personnel. With parental or caregiver permission, the school will provide child and parent contact details to the researcher to facilitate follow-up and offer the opportunity for parents or caregivers to ask questions about the study. Children with signed parental consent will be invited to participate in the study and if they assent they progress to screening. Children will be included if they are:

- Aged 11–13 years.
- Able to converse in basic English.
- A mainstream student (i.e. no intellectual disability).

Children will be excluded if their self-, teacher- or parent-report indicates suicidal or para-suicidal thoughts/behaviours or if they are already involved with secondary mental health services to address anxiety or depression. These children will be given information about relevant support services in their area and a referral made where appropriate.

### Intervention

The intervention is a manualised, occupational therapy group intervention: Kia Piki te Hauora (Uplifting our Health and Wellbeing). The intervention is designed to use engagement in developmentally appropriate activities to promote mental health and wellbeing by enabling students to understand the relationship between what they do and how they feel/think; to understand how activities in which they engage influence their identity, self-concept, health and wellbeing; to practice and develop strategies for overcoming difficult emotions; and to apply this knowledge in building and designing healthy routines, behaviours and habits in their day-to-day life that support self-esteem and participation (Table 1). Group content includes: didactic activities (e.g. occupational analysis, fight-or-flight, relaxation techniques); peer exchange (e.g. occupational charades, brainstorming); direct experience (e.g. relaxation, games); and personal exploration (e.g. occupational analysis, pepeha^a^, activity scheduling). The intervention will run for 1 hour a week over a period of 8 weeks of a school term. Outcomes will be measured at the individual level although the intervention will be provided in a group context.

**Table 1:** **Kia Piki te Hauora overview of content**

Session	Theme
1	Introduction
2	Sleep and rest occupations
3	Physical and active occupations
4	Communicating and occupations
5	Occupational disruption
6	Coping occupations
7	Values, identity and occupations
8	Closing and celebration

The intervention was developed following a consultation process with a panel of occupational therapists, cultural advisors, school personnel, children, the clinical experience of the first author (Tokolahi et al., 2013) and evidence of occupational therapy in different contexts. The theoretical knowledge underpinning the intervention is drawn from occupational therapy and science, interventions such as Lifestyle Redesign (Jackson et al., 1998; Mandel et al., 1999; Scott et al., 2001) and Five Ways to Wellbeing (The New Economics Foundation, 2008, 2011). Occupational therapy interventions are based on the understanding that engagement in meaningful doing is important to maintain and restore health and to enable us to develop (Gitlin, 2013). Occupational science has provided a five-step approach for developing evidence-based practice that informs our understanding of the influence of interventions on an individual and their environment from an occupational perspective (Jackson et al., 1998; Mandel et al., 1999). This study attempts to contribute to that process in the development of a preventative occupational therapy intervention for children, similar to the Lifestyle Redesign programs. The Five Ways to Wellbeing are a set of five simple, evidence-based actions that can improve wellbeing in everyday life: connect, give, notice, keep learning and get active (The New Economics Foundation, 2011). These messages have been woven into the intervention content with an approach that is intended to empower the child to be informed when making decisions about their own occupations, health and wellbeing (Kitching, 1998; Tacker & Dobie, 2008).

#### Piloting of the intervention

The intervention has been piloted in clinical practice with two schools. Information was collected about how the intervention might work within a school environment; about children’s responses to the intervention content and outcome measures being taken; and to gain stakeholder feedback. Positive feedback was received from both students and school personnel with a number of modifications made to the programme as a direct result of this process.

#### Treatment fidelity

Treatment fidelity will be scored by the facilitator using a fidelity implementation checklist based on those designed and used by Forgatch et al. ( 2005). The checklist records delivery of the intervention and student responsiveness per session. Each achieved item scores 1, giving a total possible score of 14 per group or 112 per cluster (Table 2). Participant fidelity will be measured through attendance and completion of homework tasks.

**Table 2:** **Intervention fidelity checklist**

Intervention component	Achieved (Y/N)
**Adherence**	
Objectives and plan posters up	Y/N
Key purpose identified (verbal)	Y/N
Activities conducted	Y/N
Materials used	Y/N
Key messages reviewed (quiz)	Y/N
**Duration and exposure**	
Between 55–60 minutes spent on session content	Y/N
**Quality of delivery**	
Facilitator comes prepared	Y/N
Facilitator encouraging and enthusiastic	Y/N
Explicit instructions given	Y/N
Constructive and positive feedback to students given	Y/N
Pacing and transitions effective	Y/N
**Programme specificity**	
Adheres to activities as designed	Y/N
Shows knowledge of content and intervention strategies	Y/N
**Student responsiveness**	
Most students are actively engaged or willingly compliant	Y/N
**Total score = total number of ‘Yes’s (max 14)**	

### Waitlist-control

The waitlist group will not receive any input during the parallel component of the trial and will only complete the baseline and post-intervention outcome measures. In the crossover component of the trial, the waitlist group will go on to receive the intervention as described.

### Outcome measures

**Figure 1:**
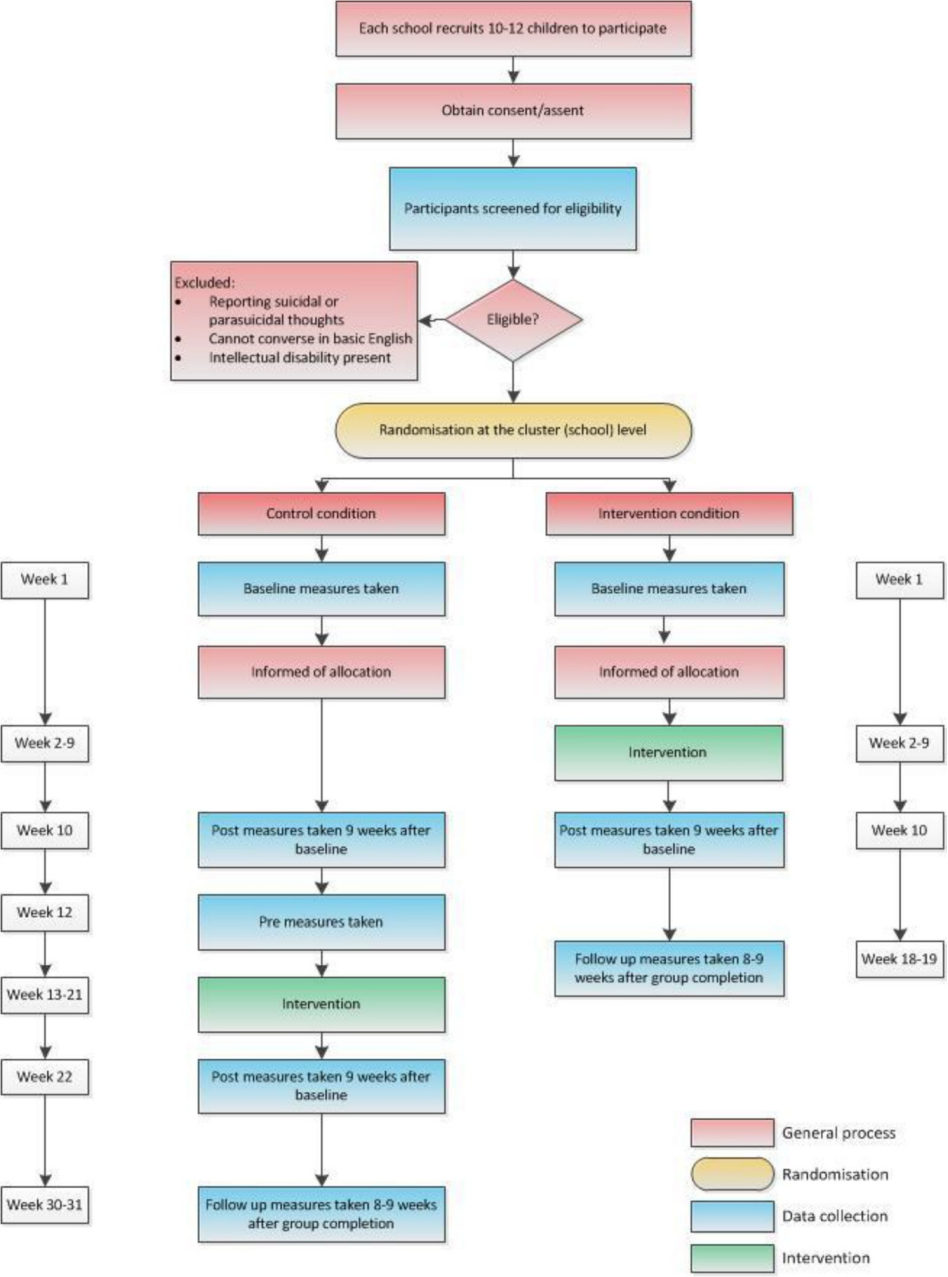
**Participant flow through the study.**

Participants will complete all outcome measures on three or five occasions, depending on the arm of the trial to which they are allocated (Figure 1).

#### Primary outcome measure

The primary outcome for effectiveness of this intervention is the participant’s self-rating of anxiety symptoms as assessed with the Multidimensional Anxiety Scale for Children – Short form (MASC-10) (Table 3). The measurement taken 9 weeks after baseline will be the primary endpoint. The MASC-10 is the most widely used measure of anxiety in research with children and its psychometric properties have been well researched. Test-retest reliability of the MASC-10 is moderate to excellent and it has good reported internal consistency (Baldwin & Dadds, 2007; March & Sullivan, 1999; Muris et al., 1998). Birmaher et al. ( 1997) have demonstrated good discriminant validity. The MASC-10 has strong discriminant validity supported by correlation with the CDI (Myers & Winters) and SCARED (Muris et al., 1998); it is sensitive to treatment effects and able to discriminate between anxious children and non-anxious children (Myers & Winters, 2002).

**Table 3:** **Outcome measures**

Outcome measure	Rater	Instrument	Description
**Anxiety**	Child	Multidimensional Anxiety Scale for Children: Short form (MASC-10) (March, 1997)	Self-rated questionnaire with 10 items measuring the child’s thoughts and emotions, specifically related to anxiety.
	Parent	Revised Child Anxiety and Depression Scale – Parent report, short version (RCADS-PS) (Weiss & Chorpita, 2011)	Parent-report questionnaire with 25 items measuring their child’s anxiety and depression.
		Modification of original measure.	
	Teacher	School Anxiety Scale (SAS) (Lyneham et al., 2008)	Teacher-rated questionnaire with 16 items that measure the level of anxious behaviours a child is displaying.
**Depression**	Child	Children’s Depression Inventory – 2 (CDI-2) (Kovacs, 1992)	Self-rated scale with 10 items measuring the child’s mood, thoughts and presentation of depressive symptoms.
	Parent	Revised Child Anxiety and Depression Scale – Parent report, short version (RCADS-PS) (Weiss & Chorpita, 2011)	Described above.
		Modification of original measure.	
**Self-esteem**	Child	Rosenberg Self Esteem Scale (RSES) (Rosenberg, 1965).	Self-rated questionnaire with 10 items measuring the child’s thoughts about their own abilities and self-worth.
	Parent	Single Item Self-Esteem Scale (SISES) (Robins et al., 2001).	Single item parent-rated questionnaire that measures the parent’s general evaluation of the child’s self-esteem.
**Participation in daily occupations**	Child	Canadian Occupational Performance Measure (COPM) - modified (Law et al., 2005)	Measures self-reported ability to participate in daily activities and satisfaction with one’s ability to participate in those daily activities. Two chosen activities the individual would like to improve on from a menu of activities pertinent to the intervention goals.
	Parent	Canadian Occupational Performance Measure (COPM) - modified (Law et al., 2005)	Measures parent-report of their child’s ability to participate in the two daily activities chosen by their child (see above) and satisfaction with their child’s ability to participate in those daily activities.
**Life satisfaction and wellbeing**	Child	Wellbeing and life satisfaction questions (The Children’s Society, 2012)	Self-rated questionnaire with five items measuring a child’s sense of wellbeing and life satisfaction.
**Knowledge about occupations, health and wellbeing**	Child	Knowledge survey	Seven sets of two questions each week in multiple choice and True/False formats. Measures change in participant knowledge of concepts and strategies covered in the intervention.

#### Secondary outcome measures

In Table 3 all secondary outcome measures are listed. Secondary outcomes include teacher- and parent-rated child symptoms of anxiety, symptoms of depression, self-esteem, participation in daily occupations, life satisfaction and wellbeing and knowledge about occupations, health and wellbeing.

#### Demographics

Socio-demographic data consisting of age, gender, ethnicity, and year of education will be collected at baseline.

### Sample size

A difference of ±5 in the *t-*score on the primary outcome measure (MASC-10) after an 8-week period can be considered clinically significant (March, 1997). To achieve a power of 80% at a significance level of 5%, 63 participants per study arm are required, a total of 126 children from 14 schools. This calculation is based on a sample size requirement for an individually randomised trial of 64 participants per arm, adjusted by a design effect of 0.98, which accounts for clustering and adjustment for baseline values. A target sample of 154 participants will be recruited to allow for possible 10% attrition.

### Randomisation

#### Sequence generation and concealment

Schools will be stratified by decile grouping and allocated unique identifiers. Within each of the three decile strata, schools will be randomly allocated to one of the two study arms (intervention or control) according to a computer-generated procedure coded by the trial statistician (fourth author), who will be unaware of the cluster identifiers. Allocation is to be revealed in three waves, after the recruitment of all participating schools is completed for a given school term. Provision for an arbitrary number of waves as needed has been made, in case recruitment is low.

#### Allocation and concealment

The randomisation scheme aims at maintaining balance within each decile stratum. The details of the scheme are otherwise withheld by the trial statistician until the end of data collection to uphold concealment. Once each school has nominated the students - and they have consented/assented to participate and been assessed for eligibility - the lead researcher will be informed of each cluster’s allocation. The lead researcher will then advise the school of the study arm to which they have been allocated, in order to facilitate logistical aspects of arranging times and venues for the intervention. Allocation will be concealed from individual participants until after completion of the baseline outcome measures.

### Blinding

Outcome measures will be taken at school by a research assistant blinded to the treatment allocation of each cluster. Two research assistants will be used to ensure they cannot guess study allocation by the number of times they have conducted testing at a school. Any inadvertent unblinding of the research assistant will be acknowledged in the final report.

Blinding of the lead researcher, who is facilitating the intervention; participants at the cluster level after screening; or the individual level after baseline measures are taken, will not be possible. This is due to the pragmatic trial design and the nature of the experimental intervention that will require active participation and interaction from participants compared to the control, which requires no involvement or participation. Blinding participants is notoriously difficult in complex and non-pharmacological interventions, by nature of them requiring significant participant interaction; the difficult is increased when the comparison group is a waitlist control group (Boutron et al., 2004). An attention-control intervention was considered to counter-act this; however, including this is beyond the scope, budget and timeframe of this study.

### Efficacy end points (EEP)

The primary EEP for this study will be one-week post-intervention on completion of the post-intervention primary outcome measure, the MASC-10. Secondary EEPs for this study will at the 8–9 week follow-up on completion of the primary outcome measure (MASC-10) and post-intervention and 8 week follow up completion of all other outcomes.

### Data management

Data will be entered into an Access database by a research assistant. Range and logic checks will be built in to assist with data cleaning. A data monitor will be assigned to review the quality and completeness of data collected. The data monitor will conduct an unblinded review of the data at two time points:

- On completion of the first round of intervention and waitlist groups being implemented (mid-July, 2014).
- On completion of the first year of the trial (January-February 2015).

This review will have the scope to inform recommendations about data quality and collection, data completeness, potential changes to the sample size and to review the adverse event log for safety issues. The lead researcher and trial statistician involved in randomisation will remain blinded to the data to prevent potential bias in intervention delivery and the blind review. No stopping rules are in place.

### Statistical analysis plan

Data will be analysed using the Statistical Package for the Social Sciences (SPSS version 20), SAS version 9.4 and R version 3.1.

### Descriptive statistics

Means, standard deviations and frequencies will be used to describe demographic, screening and outcome variables at both the cluster and the individual level. Analysis of descriptive statistics will also allow variables to be checked for any violations of assumptions underpinning the statistical techniques planned, such as checking for normality, outliers, and missing data.

### Sets for analysis

As a pragmatic trial this study will utilise an Intention-to-Treat (ITT) approach to analysis to reduce the potential for upward bias in the estimated effect size (Eldridge & Kerry, 2012). The Primary ITT data set will consist of all data from the parallel trial with participants randomised to one of the two study arms, i.e. intervention or waitlist control. Treatment allocation for this data set will be the allocated treatment at randomisation regardless of treatment received. The Secondary ITT data set will consist of all data from the Primary ITT group, plus the additional data from the crossover group (those originally allocated to the control group in the parallel trial) who go on to receive the intervention also. Treatment allocation for the Per Protocol (PP) data set will be the actual treatment received. Other major protocol violators will be removed from the PP data set.

### Subgroups for analysis

A floor effect may affect the outcomes measured on participants who present with subclinical symptoms, for whom *substantial* change is not anticipated. In order to mitigate the potential dilution of significance incurred by a floor effect, sub-groups for analysis will be defined as:

- Participants with total *t-*scores on the MASC-10 of > =56.
- Participants with total *t-*scores on the MASC-10 of <56.

### Statistical analysis

A Repeated Measures Analysis of Covariance (RM-ANCOVA) analysis of the data will allow comparisons of between-subject (intervention versus waitlist control) and within-subject (repeated measures on outcomes) factors and their interactions, while statistically controlling for baseline scores, cluster’s decile rank and other potential covariates that may be identified as the result of the blind review (e.g. gender, individual’s score on screening instrument). Incorporating individual-level covariates is more efficient (statistically) than cluster-level covariates (Eldridge & Kerry, 2012). The two predetermined covariates specified for this study are:

- Baseline scores on outcome measures;
- School decile;
- Time from baseline to assessment (treated as categorical data) for repeated measures analyses.

To maximise statistical efficiency, both post-intervention and follow-up measurements will be included in all regressions for a given outcome, with specific contrasts being used to isolate results at any time point. There is only one planned comparison for testing the primary hypothesis so no adjustment for multiplicity is required. Given the need to make multiple comparisons for the secondary hypotheses, False Discovery Rate control will be applied to protect against Type I errors (Benjamini & Hochberg, 1995).

#### Blind review

An independent statistician will review treatment data without knowledge of treatment allocation, prior to the final data analyses and after the data sets have been created. This is to finalise the statistical analysis plan. The review will have scope to change three components of the statistical plan: any additional covariates that may be included in the final analyses to enhance efficiency; transformations required or the selection of alternative models; and management of missing data. Outliers will also be identified for data verification purposes during the blind review, but not removed from the analysis sets. The decision to include additional covariates will be the result of a blind review and secondary to their allocation, as they are derived from baseline data unaffected by the independent variable.

#### Minimising loss to follow-up

Regular communication with senior school personnel will promote easier reconnection with participants at data collection points, particularly at the follow-up. Follow-up will occur 8–9 weeks post-intervention and will involve a repeat of all outcome measures in order to track any change over time. A 2-week period has been allowed for data collection at follow-up to create flexibility that is intended to improve retention. Of the four school terms, terms two and three (May-July and July-September) have deliberately been chosen for the parallel trial to increase the likelihood participants will still be affiliated with the same school at the 8-week follow-up and be contactable through that channel.

#### Missing data

This study will utilise multiple imputation for missing covariate values and a logistic regression analysis of missing data characteristics from baseline outcomes to detect any systematic patterns in missing data.

### Ethical considerations

Children are a vulnerable population and as such a number of precautions have been taken to protect their rights, including: the consent process (schools and parents must consent before children assent and all three need to agree for children to be able to participate); data confidentiality and security through de-identification; liberality of entry criteria, so children will not be excluded on the basis of their gender, socio-economic status, or relationship with the school, to ensure equitable access opportunities for all students; informed assent, whereby children are informed that participation is voluntary and that they can withdraw at any point without adverse consequences; and presence of a safety protocol in the event any children report clinically elevated symptoms of anxiety or depression.

## Discussion

This is the first known cluster-randomised controlled trial to investigate an indicated occupational therapy intervention promoting emotional wellbeing in a non-clinical sample of children. Given the lack of evidence for occupational therapy in children’s health promotion and mental health promotion generally, the results are likely to be of national and international interest. Results will contribute to the limited evidence base for occupational therapists in this field and potentially support investment in these services.

### Limitations

The intervention will be facilitated by the same clinician for all clusters. Upon ending the study, statements that the intervention - and not the clinician’s therapeutic style – were responsible for outcomes found cannot be made conclusively.

Furthermore, participants will not (and cannot) be blinded to allocation after baseline measures have been taken due to the nature of the intervention.

### Generalisability

Clusters will be drawn from a diverse, primarily urban locality; results may not be generalisable to more rural populations or locations. Generalisability will be enhanced if the sample population is representative - addressed in this study by stratifying clusters and having few exclusion criteria; through randomisation; minimising attrition; and limiting time-specific influences by conducting the trial over multiple terms in an 18 month period.

### Trial status

The present study is currently recruiting schools and participants.

## Endnote

^a^‘Pepeha’ refers to a way of introducing one’s self in Māori (indigenous people of New Zealand): in this context the pepeha has been modified to have an occupational focus and was developed in consultation with Māori cultural advisors.

## Abbreviations

CBT: Cognitive behaviour therapy
CDI: Child depression inventory
COPM: Canadian occupational performance measure
DM: Data monitor
ITT: Intention to treat
MASC: Multidimensional anxiety scale for children
RCADS: Revised child anxiety and depression scale
RM-ANVCOVA: Repeated measures analysis of covariance
RSES: Rosenberg self esteem scale
SAS: School anxiety scale
SCARED: Screen of child anxiety and related emotional disorders
SISES: Single item self esteem scale.
